# A topological data analysis-based method for gait signals with an application to the study of multiple sclerosis

**DOI:** 10.1371/journal.pone.0268475

**Published:** 2022-05-13

**Authors:** Alexandre Bois, Brian Tervil, Albane Moreau, Aliénor Vienne-Jumeau, Damien Ricard, Laurent Oudre

**Affiliations:** 1 Université Paris-Saclay, ENS Paris-Saclay, CNRS, Centre Borelli, Gif-sur-Yvette, France; 2 Université de Paris, CNRS, Centre Borelli, Paris, France; 3 Service de Neurologie, Service de Santé des Armées, Hôpital d’Instruction des Armées Percy, Clamart, France; 4 Ecole du Val-de-Grâce, Ecole de Santé des Armées, Paris, France; Edinburgh Napier University, UNITED KINGDOM

## Abstract

In the past few years, light, affordable wearable inertial measurement units have been providing to clinicians and researchers the possibility to quantitatively study motor degeneracy by comparing gait trials from patients and/or healthy subjects. To do so, standard gait features can be used but they fail to detect subtle changes in several pathologies including multiple sclerosis. Multiple sclerosis is a demyelinating disease of the central nervous system whose symptoms include lower limb impairment, which is why gait trials are commonly used by clinicians for their patients’ follow-up. This article describes a method to compare pairs of gait signals, visualize the results and interpret them, based on topological data analysis techniques. Our method is non-parametric and requires no data other than gait signals acquired with inertial measurement units. We introduce tools from topological data analysis (sublevel sets, persistence barcodes) in a practical way to make it as accessible as possible in order to encourage its use by clinicians. We apply our method to study a cohort of patients suffering from progressive multiple sclerosis and healthy subjects. We show that it can help estimate the severity of the disease and also be used for longitudinal follow-up to detect an evolution of the disease or other phenomena such as asymmetry or outliers.

## Introduction

Longitudinal follow-up and inter-individual comparison of gait trials are relevant for patients suffering with many degenerative diseases [1]. One example of such a disease is progressive Multiple Sclerosis (MS), for which gait is considered the most important source of disability [2]. Throughout this article, we will use MS as an example to illustrate our approach. Those intra/inter-individual comparisons are usually performed using semi-quantitative clinical scales, but those have limitations. In the case of MS, several clinical scales exist such as the Expanded Disability Status Scale (EDSS) [3], the Multiple Sclerosis Walking Scale-12 [4], and the Fatigue Impact Scale [5, 6]. In this study, severity of the disease was evaluated using the EDSS, which is a score from 0 to 10, ranging from normal neurological examination (0) to total impotence (9.5) or even death (10) by increments of 0.5. Those scales provide semi-quantitative or qualitative criteria for disease follow-up but they have been criticized for several reasons, including a lack of objectivity and sensitivity to clinical evolution [7–12]. This motivates the use of quantitative methods.

Over the past few years, gait quantification was made easier by the development of light, affordable inertial measurement units (IMUs). IMUs are portable systems integrating accelerometers, gyroscopes, and magnetometers that allow the synchronized measurement of linear accelerations and angular velocities in one single light, low-cost device [13]. Standard features such as velocity, step time or step length can be extracted from IMU signals. They have been used to discriminate healthy subjects from patients, or groups of patients with different levels of disease severity, but those studies rely on long protocols (walking for several minutes to get a high number of steps) [14–16] and/or gait event detection [14, 15, 17–20]. Long protocols are incompatible with patients with severely altered gait who have trouble walking a few meters, and are more difficult to include in clinical day-to-day practice. Gait event detection is either performed using expensive equipment (pressure sensitive mats, motion capture) or complex algorithms. However, those complex algorithms are difficult to apply to gait signals from pathological subjects with severely altered steps. For example, in step detection, the error on the detected start/end time of steps is typically around 10 ms for healthy subjects (HS) [21] and around 100 ms for severely affected MS patients [22]. Moreover, the above studies do not perform comparisons between different trials of the same subjects at different dates, in which case changes can be more subtle depending on the progression of the disease. This raises the question of how to compare gait trials, especially when some of them are from pathological subjects.

The goals of this article are to present a method to compare pairs of gait trials, visualize the results of all the comparisons and interpret them. Our approach is based on *Topological Data Analysis* (TDA), which we use to define a distance between gait signals, allowing us to compare gait trials. We then use a visualization algorithm to represent each trial as a 2D-point and compute features to study the structure of the obtained point cloud. By dividing the point cloud into different groups, our method makes it possible to perform both global studies to find differences in gait for different levels of severity of the disease, and longitudinal studies about the evolution of patients’ gait in time. TDA is a set of techniques derived from algebraic topology, which allows to analyze the structure of data by looking at it at different scales, and to describe the evolution of their arrangement. (see [23, 24] for a detailed introduction). The main idea behind TDA is that data are a finite subset of samples of an underlying mathematical set, whose structure can bring useful information about the system under study. For instance, a gait signal is represented by a time series, i.e. a uniform sampling of a continuous 1-dimensional physiological signal. In this setting, one of the main TDA techniques, so-called *persistent homology*, can be used to study the underlying continuous signal through the finite time series. TDA has been applied to time series in medicine and biology since the 2010s. Applications include the study of cardiac arrythmia with electrocardiograms [25], motor learning with fMRI data [26, 27], gene expression time series [28], wheeze in breathing signals [29], epileptic seizures with electroencephalograms [30], the spread of COVID-19 [31] and autism spectrum disorder [32].

TDA has been applied to the study of locomotion through time series. Motion capture data has been analyzed using TDA to model bipedal walking [33] or to perform action recognition (classification between dance, jump, run sit and walk) [34]. It has also been used to study degenerative diseases by performing binary classification of time series of gait parameters (stride, stance, and swing time) between healthy and pathological subjects (suffering from either Parkinson’s disease, Huntington’s disease or Amyotrophic lateral sclerosis) [35, 36], multi-class classification of ground reaction force time series to assess the severity of Parkinson’s disease [37], or detection of freezing-of-gait episodes [38]. To the best of our knowledge, TDA was mainly used to produce features that were fed to machine learning algorithms (such as SVM, random forest, nearest neighbors or deep neural networks) [25, 32, 34–39]. Topological features increased their performance, but they are more difficult to interpret than traditional ones (such as, for the study of locomotion, speed, step length, step time etc…) so the interpretability of the methods used in those articles is not studied. In this article, we propose an interpretable TDA-based method to compare gait trials. More precisely, we use objects from TDA to represent gait trials as points in a space in which a distance can be defined. This distance can be interpreted in terms of signal oscillations and used to compare gait trials. We applied our method to study a cohort of healthy and pathological subjects as a whole, and performed both inter-individual and intra-individual comparisons. In addition, the method has the advantage of working for time series measured with light, affordable IMUs during a protocol used in clinicians’ day to day practice.

In the first section, we describe the protocol applied to construct our dataset, introduce the method and its applications, and describe the mathematical concepts required to understand it. In the second section, we present the results of the application of our method to study MS. In the third section, we analyze and discuss those results.

## Materials and methods

### Protocol and data

Our dataset is composed of gait trials from 22 MS patients and 10 young HS. The studies involving human participants were reviewed and approved by Protection des Personnes Nord Ouest III (ID RCB: 2017-A01538–45). The patients/participants provided their written informed consent to participate in this study. The protocol is a walking exercise consisting in a 12m walk with a U-turn while wearing 4 XSens^®^ sensors (XSens^®^ Technologies, Enschede, the Netherlands; autonomy 6 h, device dimension 47 × 30 × 13 mm, weigth 16 g, acceleration range ±160 m/s^2^, angular velocity range ±2000 deg/s, dynamic accuracy roll/pitch 0.75 deg RMS, dynamic accuracy heading 1.5 deg RMS): one on the dorsal part of each foot (left foot: LF, right foot: RF), one on the lower back (T) and one on the head (H), fixed using a Velcro band designed by XSens^®^. Additional measurements including average walking speed were done for each trial using information from a GaitRite^®^ mat, which detects the initial and final contacts of the feet on the ground. The experiment was conducted in two sessions, 6 months apart, that will be referred to as M0 and M6. During each session, the protocol was performed twice. The part of the signals corresponding to the U-turn was automatically removed (using the GaitRite^®^ data, as the U-turn happened outside the mat) so that each exercise gives two signals: one for the forward path and one for the return. To sum up, for each IMU (LF, RF, T, or H) of each subject, there are 8 trials: 4 for M0 (F1: forward 1, R1: return 1, F2: forward 2, R2: return 2) and 4 for M6 (F3, R3, F4, R4).

**Fig 1:**
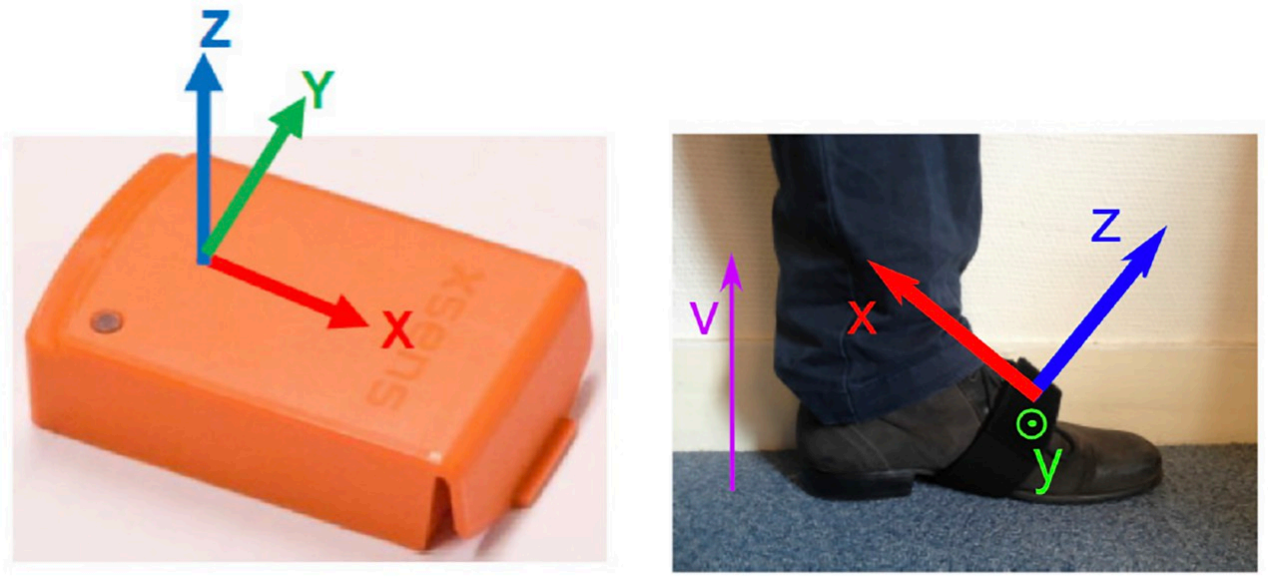
XSens sensor, with axes orientations. Source: [21].

XSens^®^ sensors are inertial measurement units (IMUs) that measure the 3D accelerations, 3D angular velocities and 3D magnetic fields. The axes are defined on Fig 1: the Y-axis is parallel to the ground and orthogonal to the walking direction. The data were sampled at 100Hz. For our study, we used the angular velocity around the Y-axis (Gyr-Y) from the feet IMUs (LF, RF), which provides signals suitable for gait assessment as it corresponds to the rotation of the foot around the medio-lateral axis. [21, 40–43]. In what follows, we will refer to those Gyr-Y signals as *gait signals*.

All the subjects were recruited between June and September 2018 at Percy Hospital (Clamart, France). The characteristics of the subjects are displayed in Table 1. Seven out of the 22 participants had an advanced disease requiring permanent walking aid (cane(s), walker and/or human help). Two patients needed human help to perform the walking test. Included participants in the MS group had an EDSS between 2 and 6.5, as disabilities greater than 7 completely impede walking.

**Table 1:** Characteristics of the subjects.

	MS (n = 22)	HS (n = 10)
**Sex (M/F)**	9/13	4/6
**Age (years)**	58 (11)	26 (1)
**Height (m)**	1.71 (0.09)	1.72 (0.09)
**Weight (kg)**	71.2 (16.6)	58.2 (10.9)
**BMI (kg/m2)**	24.3 (5.1)	21.0 (3.0)
**EDSS**	5.0 [3.5–6]	-

Baseline characteristics of patients with multiple sclerosis (MS) and healthy subjects (HS). For the age, height, weight and body mass index (BMI), the mean and the standard deviation (SD) are displayed. For the Expanded Diseases Status Scale (EDSS) the statistics are reported as median and interval quartile range (IQR).

**Fig 2:**
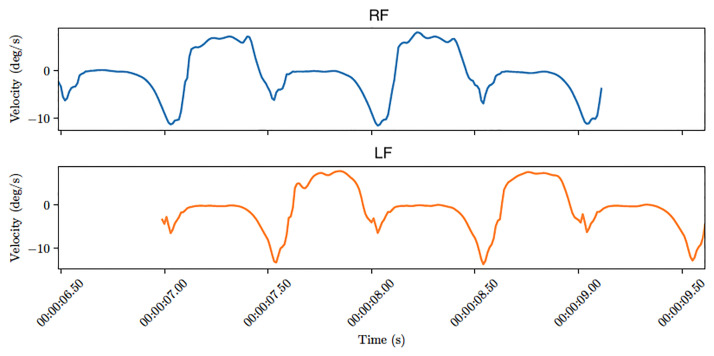
Two gait signals from a healthy subject. Top: right foot. Bottom: left foot.

**Fig 3:**
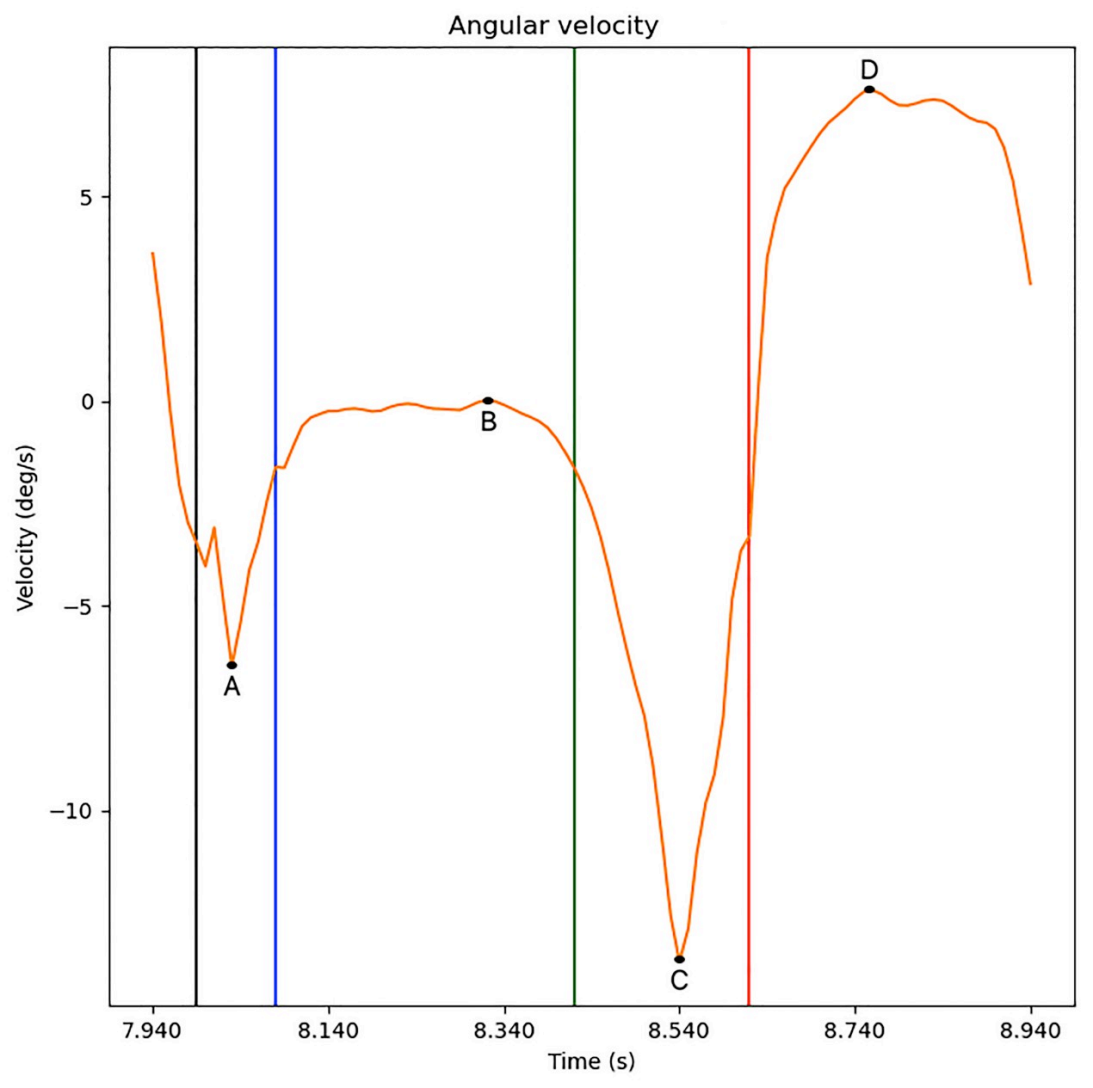
Key events of the support phase of a gait cycle. The events are represented by vertical lines. From left to right: Heel Strike (black), Foot Flat (blue), Heel Off (green) and Toe Off (red). Pairs of local extrema (A, B) and (C, D) define bars in the signal’s persistence barcode.

Fig 2 shows an example of gait signals for both feet of a healthy subject. Gait signals can be described as a succession of gait cycles, which are composed of a support phase (when the foot touches the ground) and an oscillation phase (when it is off the ground) [21]. The support phase starts with the *heel strike* (when the foot hits the ground) and ends with the *toe off* (when the foot leaves the ground) as shown in Fig 3. The plateau around 0 for angular velocity corresponds to the phase between the *foot flat* and the *heel off* events. During the oscillation phase, the angular velocity goes up, stays almost constant and then decreases until the next heel strike.

### Overview of our method and guidelines for the clinician

Here, we give an informal description of the objects used in our method and explain how we use them, followed by guidelines on how clinicians can use our method. The mathematical concepts corresponding to terms in bold will be described in the following sections.

#### Overview of our method

The goal of our method is to produce a quantitative analysis of a database of gait signals, by using comparisons based on topological properties. Given a database of gait signals and a partition of those signals into groups, it outputs a 2D point cloud and a list of features for each group of the partition.

We start by constructing a topological summary of each signal called a **persistence barcode**. A persistence barcode is a set of bars that represents the oscillations of a signal, where a long bar corresponds to a large variation. Each signal is represented by its barcode. There exists a notion of distance between barcodes called the **bottleneck distance**, that we use to compare pairs of signals via their barcodes. Before computing the bottleneck distance, we remove the longest bars from each barcode: if the barcode corresponds to a trial with *k* steps, we remove the *k* longest bars. This makes the distance less sensible to the number of steps and more sensible to the oscillations of the signal (we explain why in the mathematical description).

After having computed all the distances between pairs of barcodes, each barcode is represented as a point in the 2D Euclidean space using a **dimension reduction algorithm** called **UMAP**. This algorithm outputs a 2D point cloud whose structure is as close as possible to the structure of our set of barcodes endowed with the bottleneck distance. The obtained point cloud is a visualization of all gait signals arranged based on their topological similarity. For a given partition of the dataset, each point can be colored according to its group in order to visualize the groups on the point cloud. For each group, we compute three features: its **silhouette score** with respect to other groups (to measure their separability), and its **mean squared distance** and **diameter** (to measure its density).

**Fig 4:**
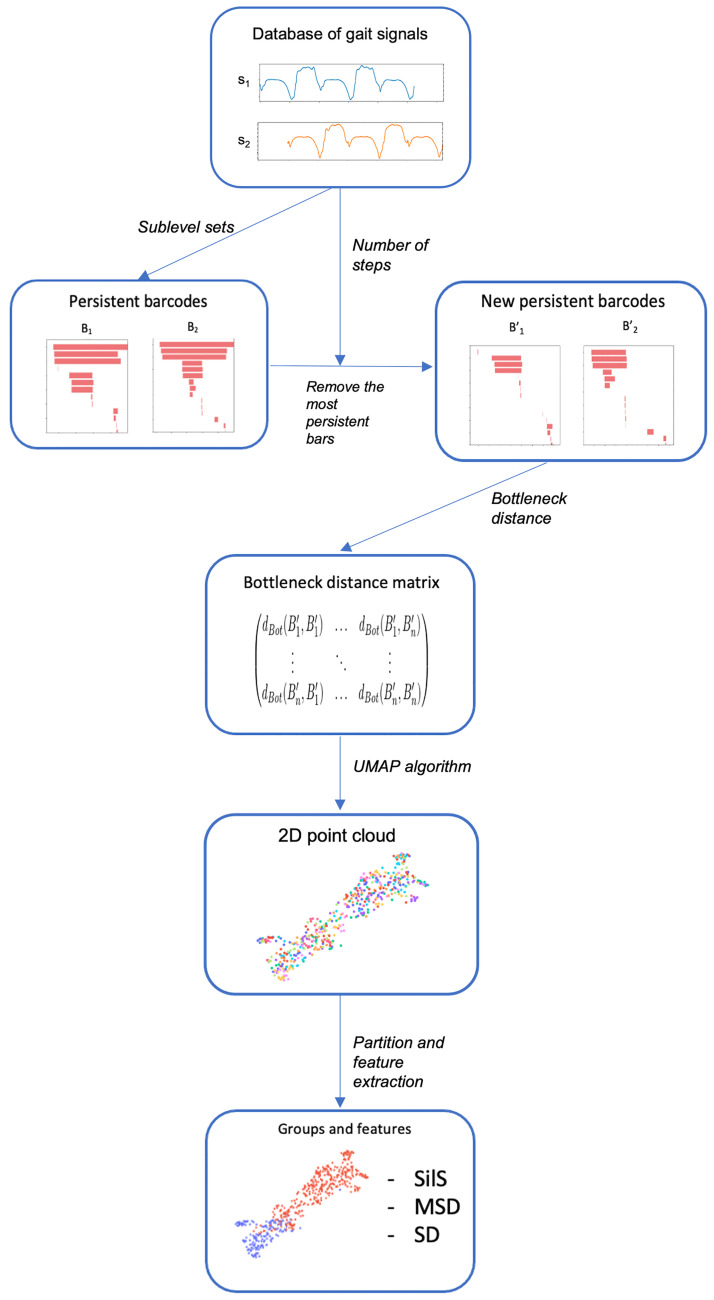
Summary of our method.

Our method can be summarized as follows (also see Fig 4):

- **Input**: a database of gait signals and partitions into groups.
- Construct the persistence barcode from all the gait signals.
- For each signal, count its number of steps *k* and remove the *k* longest bars from its barcode.
- Compute the bottleneck distance between all pairs of those barcodes.
- Compute a 2D (or 3D) point cloud using UMAP.
- **Output**: the point cloud and, for each partition, the silhouette score, mean squared distance, and squared diameter for all (pairs of) groups.

#### Guidelines for the clinician

Here, we give guidelines on how to concretely use our method to study cohorts of patients. For users who do not want to dive into the mathematical details, all the intermediate steps of the above summary of the method can be considered to already be implemented.

- Construct a database of gait signals (preferably with information on the number of steps, if not, compute it automatically as we explain later).
- Recover the point cloud.
- Partition the cohort into groups based on additional information (clinical scales, healthy/pathological, different sessions, right/left foot etc…). Each partition is defined to study a specific aspect of the cohort.
- For each partition: color points belonging to different groups in different colors, and recover tables containing all the features (silhouette scores, mean squared distances and squared diameters, or others if needed).
- Interpret (more details below).

To interpret the results, the key idea is that the relative distances between points on the point cloud reflect the difference of structure of the oscillations of the signals.

For a given partition, if two groups are well separated on the point cloud, then the criteria that define the groups are related to the differences of gait trials. For example: if the points from the M0 session of a given patient are well separated from the M6 points, then there has been a significant change in the patient’s gait and the clinician can interpret it as an evolution of the disease. On the contrary, if points of both session are not separable and form one dense group, then there is no intra/inter-session variability. If healthy subjects are well separated from patients, then the disease has an impact on gait. If points from a same session of a subject have a large mean squared distance (compared to other subjects), this means that there is a high intra-session variability. If points representing signals from the IMU placed on the left foot of a subject are well separated from those representing right foot signals, then there is an asymmetry.

We followed those guidelines in our study, which we describe in the last sections of this article.

### Mathematical description our of method

This section describes the mathematical construction of the objects from TDA used in our method.

#### Persistence barcodes from sublevel sets

We now explain how to perform TDA on time series using sublevel sets. For a given real-valued function *f*: *t* ↦ *f*(*t*) and threshold α∈R, the sublevel set *F*_*α*_ is defined as

$$\begin{array}{c} F_{\alpha }=f^{-1}([-\infty ,\alpha ]). \end{array}$$ (1)

As explained above, our goal is to study the evolution of the arrangement of data through different scales. This evolution can be summarized by a so-called *persistence barcode*. Formally, the persistence barcode (from sublevel sets) of a signal described by a function *f* is the set of pairs (date of birth, date of death) of the connected components of the sets *F*_*α*_ as *α* goes from −∞ to + ∞. That is to say, for a given *α*, if *F*_*α*_ has a connected component with no point belonging to any *F*_*β*_ such that *β* < *α*, we say this component was born at *α*. If two components from *F*_*β*_, *β* < *α* have merged in *F*_*α*_ then we say that the youngest one died at *α*.

**Fig 5:**
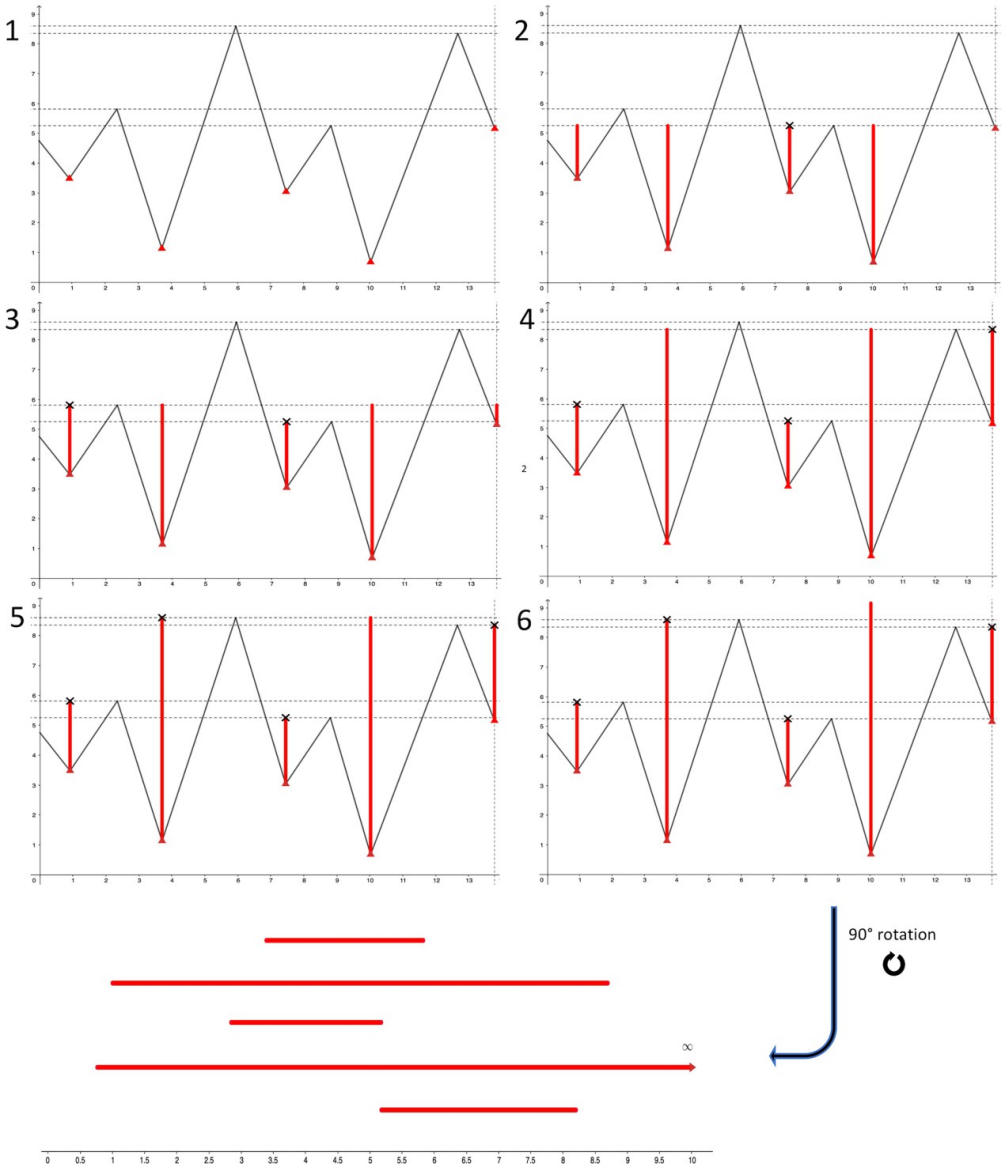
Construction of a persistence barcode. 1: Mark all the local extrema except for the first maximum. The minima are marked by red triangles, the maxima by horizontal lines. 2: Grow bars until the first local maximum. The third bar stops growing. 3: At the second local maximum, the first bar stops growing. 4: At the third local maximum, the fifth bar stops growing. 5: At the fourth local maximum, the second bar stops growing. 6: The fourth bar grows to infinity. Bottom: Horizontal representation of the persistence barcode.

**Fig 6:**
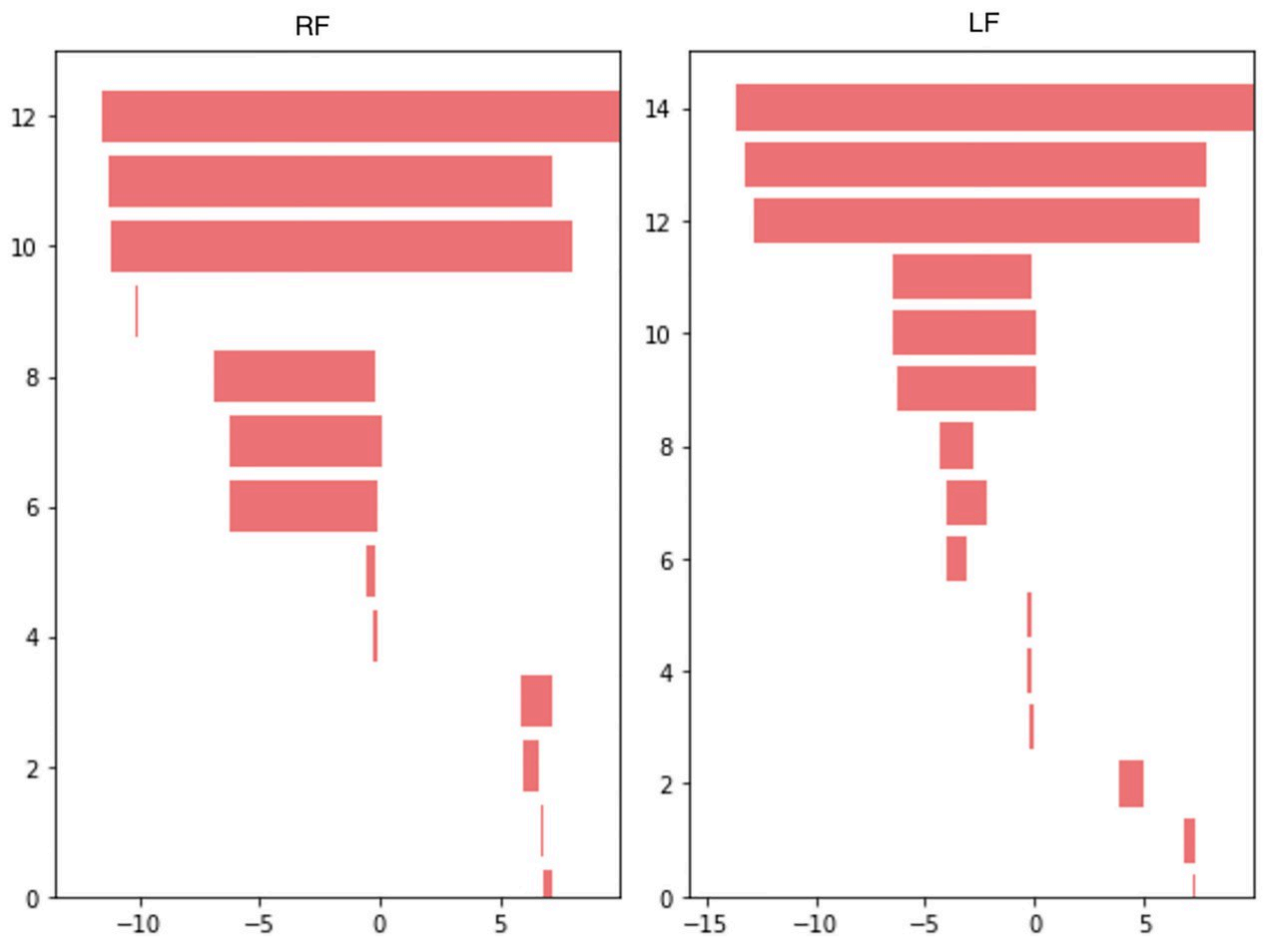
Persistence barcodes from the sublevel sets of the signals from Fig 2.

The persistence barcode of the sublevel sets of a time series can be constructed by pairing local minima to local maxima using the following algorithm (illustrated in Fig 5):

1. Mark the level on the Y-axis of all the local extrema of the signal. The first and last points can be ignored if they are local **maxima**.
2. Start drawing a vertical bar going up from the **global minimum**.
3. Each time the bars reach the level of a another **local minimum**, start another vertical bar at this minimum. Then make all the bars go up to the level of the next extrema.
4. Each time the bars reach the level of a **local maximum**, if that point has one bar at its left and one at its right, then the shortest of those two bars stops growing. Then make all the bars go up to the level of the next extrema.
5. When the bars reach the **global maximum**, stop, as the remaining bar will keep growing indefinitely.
6. The persistence barcode is made of all the pairs of (start, end) vertical coordinates of the bars obtained this way (we ignore time coordinates), where the longest bar goes up to + ∞. It is usually represented horizontally as in Fig 6. This representation is obtained by keeping only the bars and Y-axis, and rotating the graph by 90° clockwise.

Let us now describe the persistence barcode corresponding to a single gait cycle. Persistence barcodes of time series can be understood in terms of pairs of local minima and maxima. On Fig 3, four important local extrema can be noticed:

- One minimum between the heel strike and foot flat (point A on Fig 3). The angular velocity keeps decreasing for some time after the heel strike before going back to zero at foot flat.
- One maximum around zero at the plateau between foot flat and heel off (point B on Fig 3).
- A second minimum just before toe off (point C on Fig 3). Heel off makes the angular velocity decrease below zero and toe off makes it go back above zero.
- A second maximum during the oscillation phase at the high plateau of the gait cycle (point D on Fig 3).

Let (*t*_*P*_, *y*_*P*_) denote the coordinates of a point *P* of a time series. The barcode of the signal on Fig 3 will have two bars that are characteristic of gait cycles: a long bar (*y*_*C*_, +∞) corresponding to the pair (C, D) and a smaller bar (*y*_*A*_, *y*_*B*_) corresponding to (A, B). The other smaller bars are considered to be oscillations, irregular movements or noise.

Let us now consider full gait trials. Fig 6 shows the persistence barcodes of the two typical gait signals represented on Fig 2. Three gait cycles can be distinguished for both signals on Fig 2, each one is responsible for a long bar and a medium-sized bar on Fig 6. Note that only one bar goes to infinity, any other bar corresponding to a pair (P, Q) has coordinates (*y*_*P*_, *y*_*Q*_). The smaller bars are oscillations. For example, on the LF barcode, the 7^th^, 8^th^ and 9^th^ bars (counting from top to bottom) correspond to the oscillation that happens just after heel strike during each gait cycle (Fig 3 shows where the heel strike happens on a signal). Those oscillations are less present on the RF signal.

Note that counting the longest bars is equivalent to counting the steps (including the last step, that may be incomplete): on Fig 6, the three long bars correspond to the three steps visible on Fig 2.

#### Distance between barcodes

To compare persistence barcodes, a distance called the *bottleneck distance* can be used [23]. Recall that a barcode is a set of pairs (*x*, *y*) that are the start and end vertical coordinates of each bar. The same pair can be represented multiple times and *y* can be equal to +∞ (this happens exactly once if the signal is defined on an interval). For barcodes *B* and *B*′, the bottleneck distance is based on an idea from optimal transport, using bijections between the two barcodes (functions from *B* to *B*′ such that each bar from *B*′ is associated to a unique bar from *B*). Let *Γ*(*B*, *B*′) be the set of bijections from *B* to *B*′. Note that if two finite sets do not have the same number of elements there are no bijections between them, so we include to *B* and *B*′ all bars (*x*, *x*) of length zero (an infinite number of times), so that there always exists a bijection between *B* and *B*′. For any *γ* ∈ Γ(*B*, *B*′) and any bar *b* = (*x*, *y*) ∈ *B* such that *γ*((*x*, *y*)) = *b*′ = (*x*′, *y*′), the two bars can be compared using the infinite norm:

$$\begin{array}{c} {\|b-b^{\prime }\|}_{\infty }=\{\begin{array}{cc} |x-x^{\prime }| & \text{if}\;y=y^{\prime }=\infty \\ max(|x-x^{\prime }|,|y-y^{\prime }|) & \text{otherwise.} \end{array} \end{array}$$ (2)

For each *γ*, the pair of bars (*b*, *b*′) such that ‖*b* − *γ*(*b*)‖_∞_ is maximal gives a notion of similarity between *B* and *B*′ induced by the pairing of bars defined by *γ*. The bottleneck distance is defined by choosing the bijection that minimizes this quantity (which means that we associate each bar of *B* to the one in *B*′ that is the most similar). Formally, the bottleneck distance between *B* and *B*′ is given by:

$$\begin{array}{c} d_{Bot}\left(B,B^{\prime }\right)=\underset{\gamma \in \Gamma (B,B^{\prime })}{inf}\;\underset{b\in B}{sup}\;{\left\|b-\gamma \left(b\right)\right\|}_{\infty }. \end{array}$$ (3)

Stability theorems [44–47] prove that under generic assumptions, barcodes associated with similar signals are close for the bottleneck distance.

As explained above, the number of long bars in barcodes from gait signals is the number of steps. This implies that the bottleneck distance between two barcodes corresponding to trials with a different number of steps will be high. Indeed, in that case, one of the two barcodes will have more long bars than the other so each bijection *γ* will pair at least one long bar *b* to a short bar *γ*(*b*), resulting in a high ‖*b* − *γ*(*b*)‖_∞_ and thus in a high distance.

This means that the bottleneck distance will mainly distinguish signals that have a different number of steps. However a different number of steps can be due to many factors such as experimental conditions, the subject’s height, age, or the foot that does the first step (a RF and a LF signal from the same exercise can have a different number of steps if a subject starts and ends with the same foot). To reduce this step-counting effect and focus more on oscillations, we propose to count the steps on each signal and remove the *k* longest bars from the corresponding barcode, where *k* is the number of steps. The number of steps can be computed from signals using the *autocorrelation function* (ACF) of each signal. The time when the second peak of the ACF is reached is the duration of the first gait cycle, and the number of steps can be deduced from this quantity and the duration of the trial. This method is heuristic and has limitations, notably with signals from patients with very deteriorated gait. For any future clinical use of our method, steps could be counted during the protocol and included in the data.

Once the barcodes from every gait signal of the database have been computed, the next step of our method is the following: for each pair of barcodes *B* and *B*′ corresponding to trials with respectively *k* and *k*′ steps, remove the *k* (resp. *k*′) longest bars from *B* (resp. *B*′) to get a new barcode $\tilde{B}$ (resp. $\tilde{B^{\prime }}$) and compute $d_{Bot}\left(\tilde{B},\tilde{B^{\prime }}\right)$.

#### Visualization algorithm

Barcodes (and the gait signals they represent) can be seen as points in a (non-Euclidean) metric space, endowed with the bottleneck distance. To be visualized, these points need to be projected onto the 2D or 3D Euclidean space. Several algorithms can compute such a projection, including the UMAP algorithm [48], t-SNE [49] and multidimensional scaling (MDS) [50]. MDS focuses on respecting the distance matrix, while UMAP and t-SNE intend to represent the structure of the original metric space. We chose UMAP to focus on structure, and because its parameters can be chosen so that more global structure is preserved than with t-SNE.

The UMAP algorithm has been used in applications such as the study of odors and molecular structures [51], physical and genetic interactions [52] or genomic data [53].

The UMAP algorithm takes as input a distance matrix (here, the matrix of all bottleneck distances between all pairs of barcodes) and two parameters: n_neighbors and min_dist. It outputs a 2D (or 3D) point cloud where each point represents a gait signal, whose structure induced by the Euclidean distance is as close as possible to the structure induced by the bottleneck distance on the space of barcodes. That is to say, if two barcodes are close according to the bottleneck distance, then the corresponding points in the point cloud will be close according to the Euclidean distance.

The two parameters control the compromise between respecting the local and global structure of the data. A low n_neighbors parameter makes the UMAP algorithm focus more on the local structure around each point, whereas a high n_neighbors will make it focus on the global structure. The min_dist parameter is the minimum distance allowed between two points in the point cloud. A low min_dist allows the algorithm to represent similar barcodes as close points in the point cloud. A high min_dist will prevent it to produce very dense neighborhoods to make the global structure appear more clearly.

**Fig 7:**
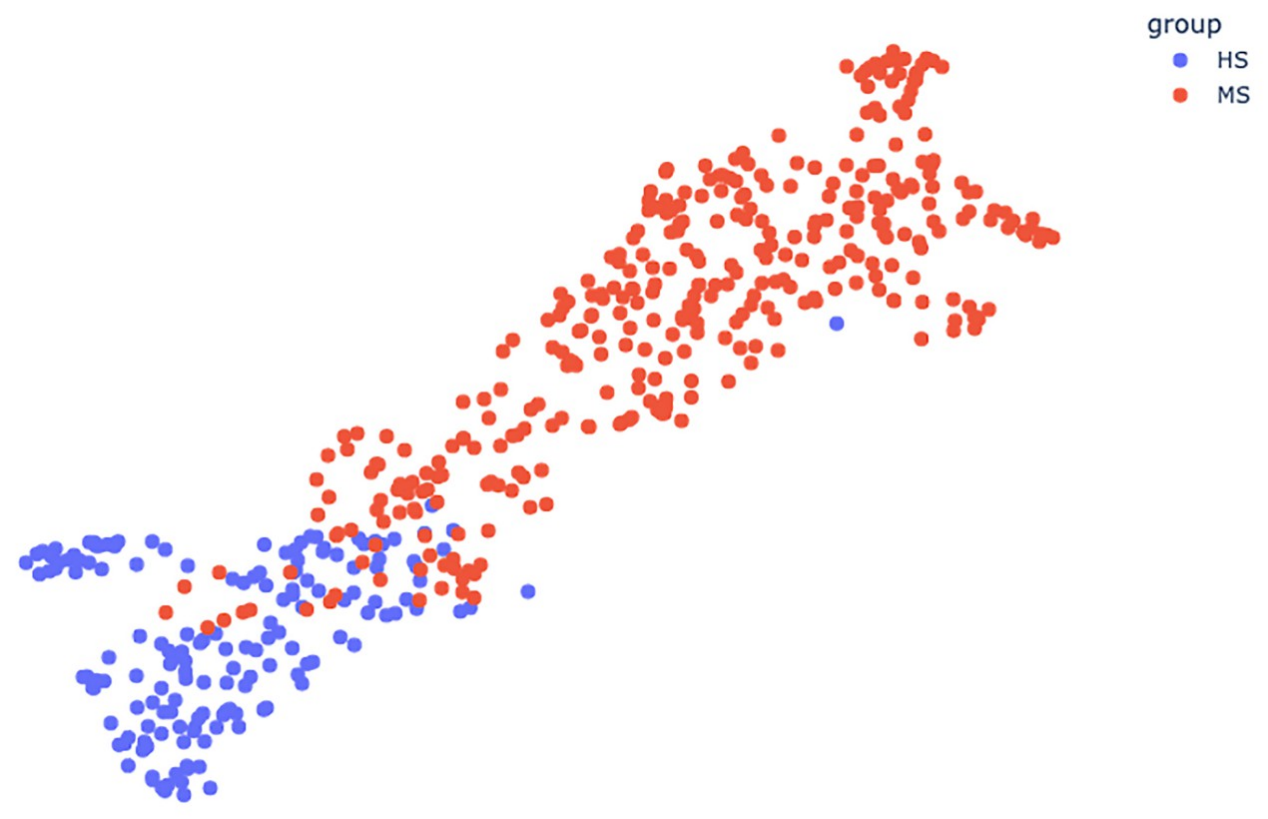
UMAP plot colored by group.

In what follows, we use UMAP with the metric induced by the bottleneck distance, with parameters n_neighbors = 45, min_dist = 0.3 and in 2D. See Fig 7 for an example. Interactive versions of the plots are provided with this article.

### Feature extraction

The goal of our method is to study a database of gait signals. As the UMAP projection preserves the structure induced by the bottleneck distance between barcodes, information can be extracted by studying the relative positions and neighborhood relations of points in the point cloud. To do this, we regroup signals that share a given characteristic (for example: both being from a healthy/pathological subject, or from the same session) and study the geometry of the groups and the relations between groups, using three features: the *silhouette score* (Sil), the *mean squared intra-group distance* (MSD) and the *squared diameter* (SD).

Note that, when using a UMAP projection, no information can be extracted from the absolute coordinates of the points or the absolute distance between two points. The position of a point should only be studied through its distance to other points, and distances should be studied in a relative way. For example, saying that point A and point B are closer together than they are with point C means that signal A is more similar to signal B than to signal C.

Let *S* = (*s*_*i*_)_1≤*i*≤*n*_ be a database of *n* gait signals and *X* = (*x*_*i*_)_1≤*i*≤*n*_ be a (2D or 3D) point cloud obtained with the above method, where each point *x*_*i*_ represents a signal *s*_*i*_. Let (*C*_*i*_)_*i*∈*I*_ be a partition of *X* into groups.

#### Silhouette score

Let *i*, *j* be two distinct indices in *I*, |*C*| denote the cardinal of set *C* and ‖.‖_2_ denote the Euclidean norm. The silhouette score of a point *x* ∈ *C*_*i*_, with respect to group *j* is defined as:

$$\begin{array}{c} Sil\left(x,C_{j}\right)=\frac{b-a}{max(a,b)}, \end{array}$$ (4)

where $a=\frac{1}{|C_{i}|-1}\sum_{y\in C_{i},y\neq x}\|x-{y\|}_{2}$ is the mean distance between *x* and all other points in the same group, and $b=\frac{1}{|C_{j}|}\sum_{y\in C_{j}}\|x-{y\|}_{2}$ is the mean distance between *x* and all points in group *j*.

The silhouette score of group *i* with respect to group *j* is defined as the mean silhouette score of the points of group *i* with respect to group *j*:

$$\begin{array}{c} Sil\left(C_{i},C_{j}\right)=\frac{1}{|C_{i}|}\sum_{x\in C_{i}}Sil\left(x,C_{j}\right). \end{array}$$ (5)

#### Mean squared distance

The MSD of group *i* is defined as:

$$\begin{array}{c} MSD\left(C_{i}\right)=\frac{2}{|C_{i}\left|(\right|C_{i}\left|-1\right)}\sum_{x,y\in C_{i},x\neq y}\|x-{y\|}_{2}^{2}. \end{array}$$ (6)

#### Squared diameter

The squared diameter of group *C*_*i*_ is defined as:

$$\begin{array}{c} SD\left(C_{i}\right)=\underset{x,y\in C_{i}}{max}(\|x-{y\|}_{2}^{2}). \end{array}$$ (7)

Note that we have squared the distance to be consistent with the MSD.

#### Interpretation of the features

The silhouette score is a clustering evaluation metric that is used to determine if the groups we define can be considered as clusters in our point cloud. It takes values between -1 and 1. A value close to 1 means good clustering (the groups are well separated from one another and dense), a value close to 0 means overlapping groups, and a value close to -1 means bad clustering.

To understand this, let us consider concrete examples. For a point *x* ∈ *C*_1_, *Sil*(*x*, *C*_2_) = 0.5 means that *b* = 2*a* (using the same definition as above for *a* and *b*), i.e. that *x* is on average twice as far from points of *C*_2_ than from points of *C*_1_. Thus, *Sil*(*C*_1_, *C*_2_) = 0.5 means that on average a point of *C*_1_ will be twice as far from *C*_2_ than from *C*_1_. On the contrary, *Sil*(*x*, *C*_2_) < 0 means that *x* is on average closer to *C*_2_ than to *C*_1_, and having *x* in *C*_2_ would increase the score. Negative scores are thus interpreted as bad clustering. If *Sil*(*x*, *C*_2_) is close to zero, then the difference between *a* and *b* is small compared to the size of the groups, so *x* can be considered to be as close to *C*_1_ and *C*_2_, i.e. *x* is “between *C*_1_ and *C*_2_”. A small *Sil*(*C*_1_, *C*_2_) then means that the two groups are overlapping.

The fact that values are always between -1 and 1 is an advantage of the silhouette score compared to other clustering evaluation metrics because it can be interpreted on its own without necessarily being compared to the score of a different clustering. Note that the silhouette score is not symmetrical: *Sil*(*C*_*i*_, *C*_*j*_) is not necessarily equal to *Sil*(*C*_*j*_, *C*_*i*_).

The MSD and SD measure the density of the groups. They should only be interpreted relatively to other groups. The MSD measures the average (squared) distance between points of the same group, so a smaller MSD means that a group has points that are closer to one another on average. The SD measures the largest of those distances. The SD is complementary to the MSD because it focuses on the two points that are the furthest apart. For example, a group *C*_1_ of points uniformly spread on a line and a group *C*_2_ with one point at the beginning of the line and all the other points at the end would have the same SD but *C*_2_ would have a significantly lower MSD as it is very dense except for one outlier. A joint analysis of the MSD and SD can thus detect outliers in a group with relatively low MSD and high SD.

## Results

We applied our method to study the database of gait signals described above. The study is divided in three parts: the first one compares healthy subjects (HS) to multiple sclerosis (MS) patients, the second one is a series of experiments that compare subjects with different EDSS scores, and the third one studies the evolution of each subject between M0 and M6. Each experiment corresponds to a different partition of the database.

### HS/MS experiment

Here, we divide our database into two groups: *HS* and *MS*. *HS* is the group of points from healthy subjects and *MS* is the group of points from multiple sclerosis patients.

This partition can be visualized on Fig 7. Table 2 shows the Sil, MSD and SD values for the partition (*HS*, *MS*).

**Table 2:** Features for HS and MS patients.

*Sil*(*HS*, *MS*)	*Sil*(*MS*, *HS*)	*MSD*(*HS*)	*MSD*(*MS*)	*SD*(*HS*)	*MSD*(*MS*)
**0.68**	**0.41**	6.8	18.6	109.5	147.2

The subjects have been divided into two groups: HS and MS patients. The silhouette scores, MSD and SD have been computed on those groups.

The values of the silhouette score are: *Sil*(*HS*, *MS*) = 0.68 and *Sil*(*MS*, *HS*) = 0.41.

### EDSS experiments

In this section, we perform a series of experiments to study the relation between the EDSS and the relative position of points on the point cloud. For a given threshold *i*, we divide our database into two groups: {*EDSS* ≤ *i*} and {*EDSS* > *i*}. {*EDSS* ≤ *i*} is the group of points from subjects with EDSS lower than or equal to *i* and {*EDSS* > *i*} is the group of signals from subjects with EDSS strictly higher than *i*. HS are given an EDSS of 0. We consider the following values of *i*: 0, 2, 2.5, 3, 3.5, 4, 4.5, 5, 5.5, and 6 as there are no patients with EDSS under 2 or above 6.5. Note that the experiment with *i* = 0 is the HS/MS experiment.

**Fig 8:**
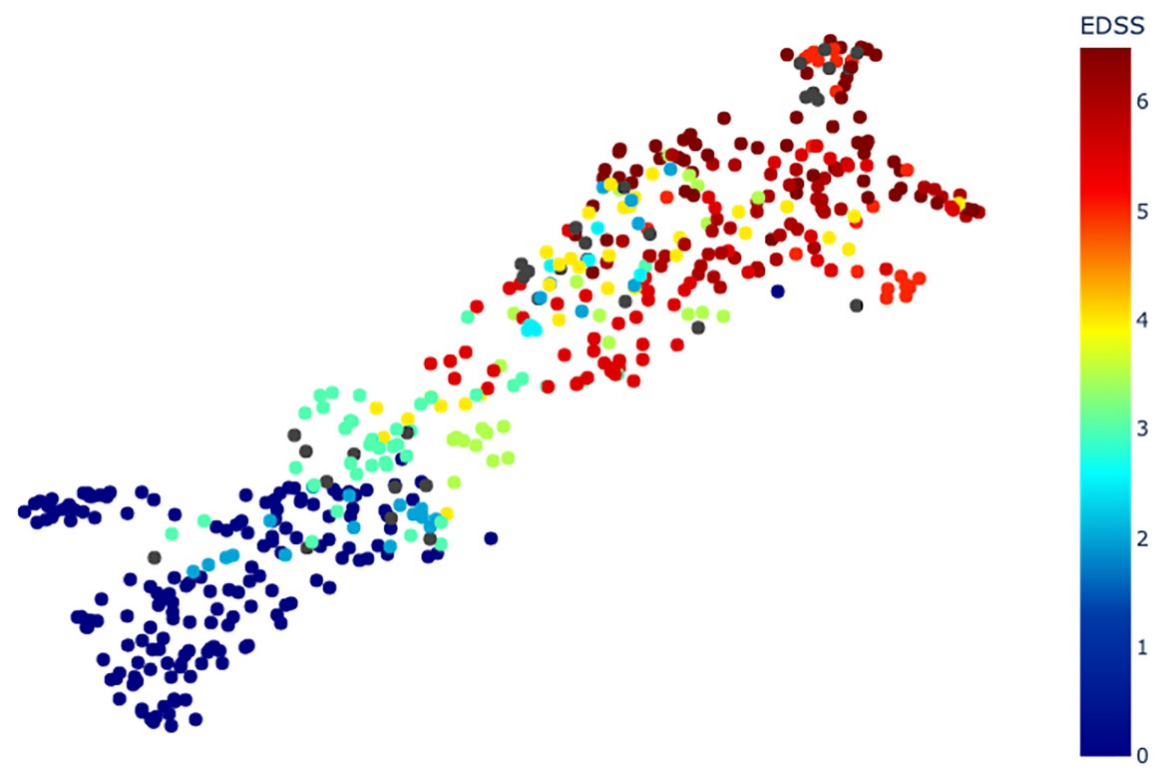
UMAP plot colored by EDSS.

The EDSS corresponding to each point can be visualized on Fig 8. Table 3 shows the Sil, MSD and SD values for each partition ({*EDSS* ≤ *i*}, {*EDSS* > *i*}). In this table, for the sake of clarity, we use the notation *Sil*(≤ *i*, > *i*) instead of *Sil*({*EDSS* ≤ *i*}, {*EDSS* > *i*}).

**Table 3:** Features for points with EDSS lower or equal to/ stricly higher than each threshold.

i	*Sil*(≤ *i*, > *i*)	*Sil*(> *i*, ≤ *i*)	*MSD*({*EDSS* ≤ *i*})	*MSD*({*EDSS* > *i*})	*SD*({*EDSS* ≤ *i*})	*SD*({*EDSS* > *i*})
0	**0.68**	**0.41**	6.8	18.6	109.5	147.2
2	**0.60**	**0.46**	11.2	16.1	136.8	137.5
2.5	**0.54**	**0.44**	13.7	16.4	136.8	137.5
3	**0.52**	**0.56**	14.0	11.2	136.8	95.3
3.5	**0.44**	**0.58**	17.6	9.9	142.0	95.3
4	**0.34**	**0.60**	22.4	8.8	168.5	57.3
4.5	**0.34**	**0.60**	22.4	8.8	168.5	57.3
5	**0.21**	**0.58**	29.9	8.5	210.3	57.3
5.5	**0.15**	**0.70**	31.2	4.8	210.3	26.0
6	**0.088**	**0.68**	34.1	4.8	210.3	26.0

For a given threshold *i*, the points from each subject have been divided in two groups: points corresponding to patients with EDSS lower than or equal to *i* ({*EDSS* ≤ *i*}), and points with EDSS higher than *i* ({*EDSS* > *i*}). The silhouette scores, MSD and SD have been computed on those groups for several values of *i*.

All silhouette scores are positive. For each *i*, at least one of the two silhouette scores *Sil*({*EDSS* ≤ *i*}, {*EDSS* > *i*}) and *Sil*({*EDSS* > *i*}, {*EDSS* ≤ *i*}) is above 0.5.

#### Result using walking velocity instead of TDA

**Fig 9:**
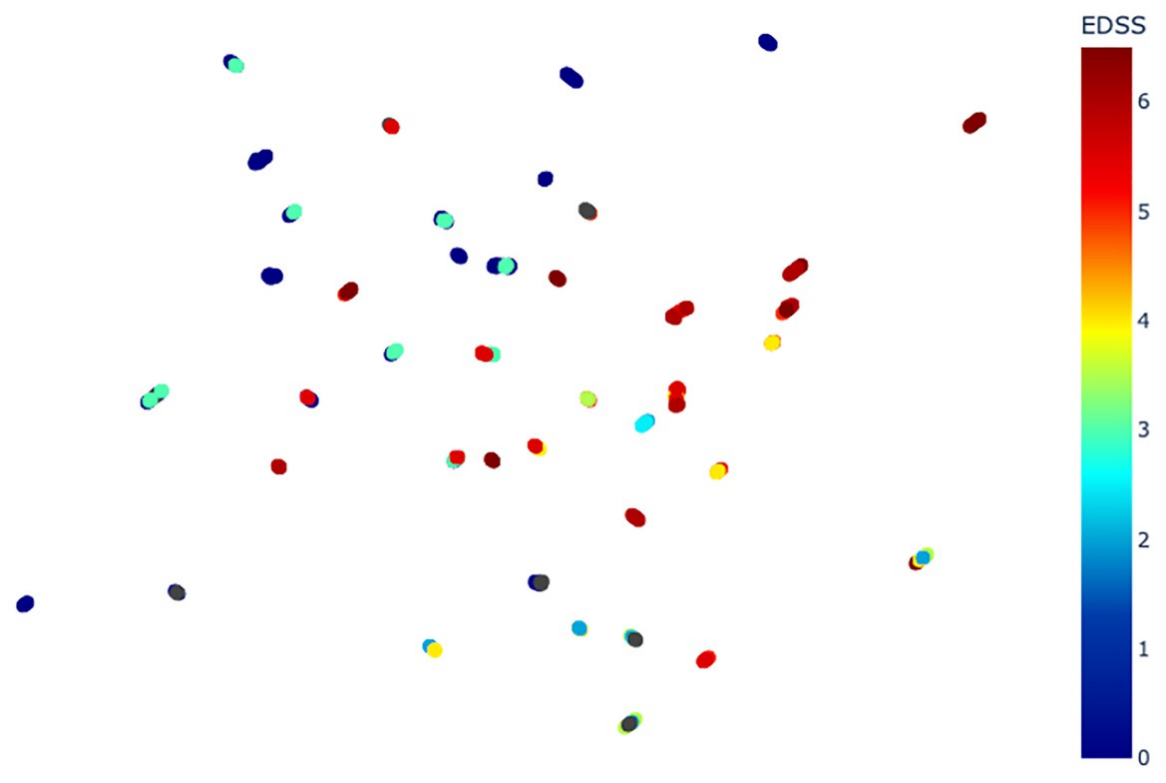
UMAP plot obtained using the difference of walking speed as a distance between signals, colored by EDSS.

Fig 9 shows the point cloud obtained by performing the same experiment except that the bottleneck distance was replaced by the difference of walking velocity (in m/s) between trials. Points are colored according to the EDSS of the corresponding subject.

### Longitudinal experiment

In this section, for each subject, we compare the M0 session to the M6 session. We start by dividing our database into 32 groups (each corresponding to one subject): each subject is given an ID between 1 and 32, and group *i* corresponds to signals from subject *i*. Then, each group *i* is subdivided into two groups *M*0_*i*_ and *M*6_*i*_. *M*0_*i*_ is the group of signals from the M0 session of subject *i*, *M*6_*i*_ is the group of signals from their M6 session. The final partition has 64 groups: {(*M*0_*i*_, *M*6_*i*_), 1 ≤ *i* ≤ 32}. The goal of this experiment is to study the evolution of each patient, therefore silhouette scores are only computed for pairs (*M*0_*i*_, *M*6_*i*_).

The partition into 32 groups and the partition into 64 groups can be visualized on the interactive plots provided with this paper (along with those corresponding to Figs 7 and 8). Table 4 shows the Sil, MSD and SD values for each pair (*M*0_*i*_, *M*6_*i*_).

**Table 4:** Individual silhouette score, mean squared distance and squared diameter for each session.

i (ID)	*Sil*(*M*0_*i*_, *M*6_*i*_)	*Sil*(*M*6_*i*_, *M*0_*i*_)	*MSD*(*M*0_*i*_)	*MSD*(*M*6_*i*_)	*SD*(*M*0_*i*_)	*SD*(*M*6_*i*_)
1	-0.17	0.19	3.1	2.1	12.1	8.9
2	-0.29	0.55	**24.9**	2.7	**99.9**	8.0
3	0.32	0.08	1.4	2.2	5.8	6.6
4	-0.03	-0.02	0.9	0.9	3.4	3.0
5	0.01	0.15	0.3	0.3	1.2	1.0
6	-0.06	0.53	1.4	0.2	3.6	0.7
7	0.28	0.40	0.9	0.7	2.7	2.7
8	-0.22	0.14	1.8	0.8	6.6	2.1
9	0.03	-0.11	2.3	3.3	5.3	12.7
10	0.31	0.22	1.9	2.9	6.2	8.7
11	0.21	-0.09	1.3	2.1	3.9	6.1
12	**0.83**	**0.96**	**1.4**	**0.1**	**5.1**	**0.3**
13	0.26	-0.14	0.2	0.4	0.6	1.0
14	0.61	0.79	1.6	0.5	6.0	1.3
15	**0.49**	**-0.21**	1.9	**9.5**	7.5	**30.6**
16	-0.07	-0.04	3.6	3.3	10.3	10.4
17	0.00	0.02	2.2	2.1	5.7	5.2
18	0.12	-0.12	2.0	3.5	4.8	9.4
19	0.42	0.21	2.3	3.3	10.8	9.1
20	0.47	0.01	0.5	2.2	1.1	8.4
21	0.38	-0.03	4.4	12.8	12.5	37.1
22	0.08	-0.09	0.9	1.1	2.9	3.8
23	0.08	0.19	2.6	1.9	7.2	5.3
24	0.62	0.30	0.6	1.4	2.2	4.7
25	-0.19	0.06	6.0	3.3	23.6	11.4
26	-0.01	0.01	2.2	2.0	8.4	7.4
27	-0.05	0.22	2.7	1.5	7.9	4.2
28	0.52	0.19	1.4	4.0	5.4	16.0
29	0.11	-0.10	2.9	3.4	7.8	14.6
30	-0.07	-0.01	3.2	3.1	7.1	8.4
31	-0.03	0.06	1.6	1.2	5.9	4.0
32	0.29	-0.30	0.5	1.8	1.7	7.2

Sil, MSD and SD values for each pair (*M*0_*i*_, *M*6_*i*_) for each subject of ID *i*.

#### Study of subject 12

The two highest silhouette scores are from subject 12: *Sil*(*M*0_12_, *M*6_12_) = 0.83 and *Sil*(*M*6_12_, *M*0_12_) = 0.96. Subject 12 has an EDSS of 4 at M0 and 5 at M6 and is the only MS patient to have a variation of their EDSS of more than 0.5.

**Fig 10:**
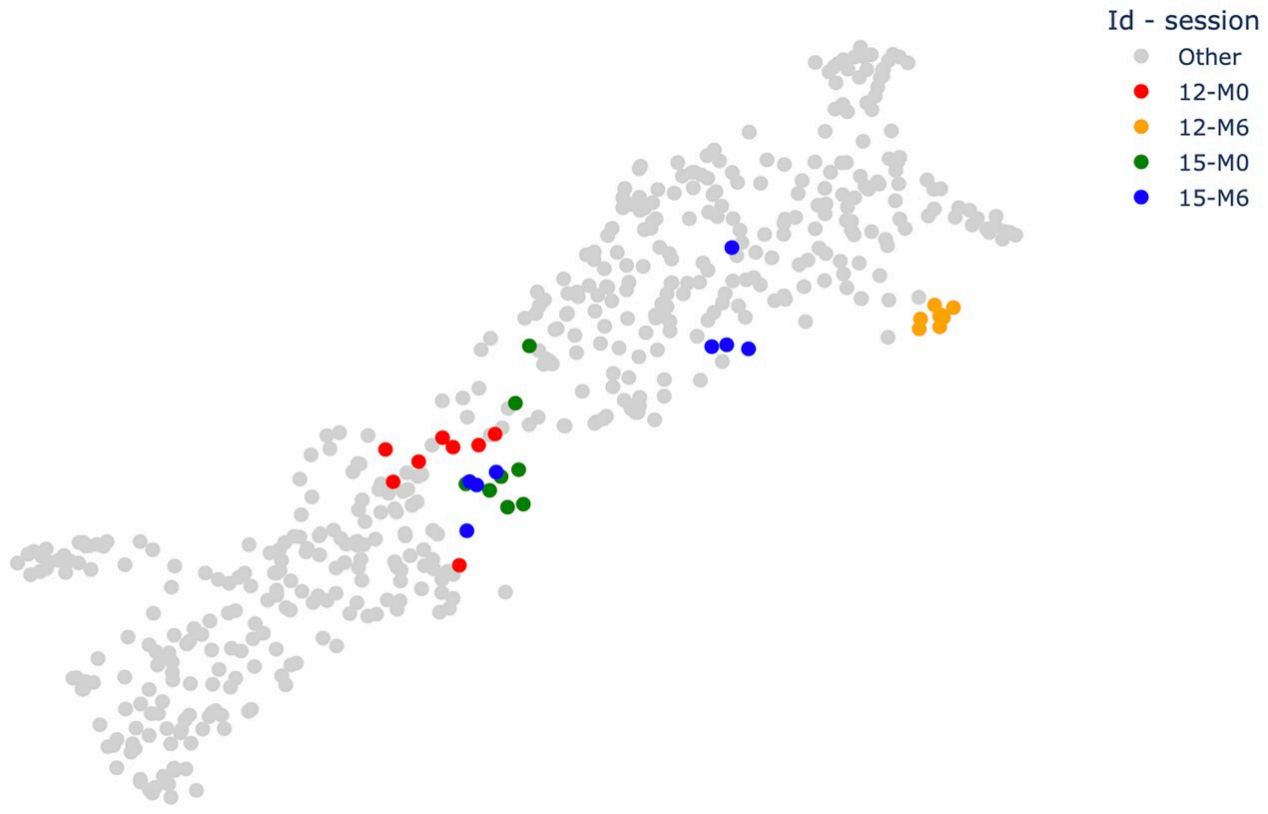
Longitudinal study of subjects 12 and 15. The point cloud is the same as on Figs 7 and 8 but colored differently. The red (resp. orange) points are the M0 (resp. M6) points of subject 12. The green (resp. blue) points are the M0 (resp. M6) points of subject 15. Grey points correspond to other subjects.

Fig 10 shows the position of points from subject 12 on the point cloud.

#### Study of subject 15

Subject 15 has silhouette scores *Sil*(*M*0_15_, *M*6_15_) = 0.49 and *Sil*(*M*6_15_, *M*0_15_) = −0.21. *M*6_15_ has the second highest MSD and SD of all M6 sessions.

Fig 10 shows the position of points from subject 15 on the point cloud.

#### Study of subject 2

The group *M*0_2_ of points from the M0 session of subject 2 has the highest MSD and SD of Table 4: *MSD*(*M*0_2_) = 24.9 and *SD*(*M*0_2_) = 99.9. The second highest MSD is about 13 and the second highest SD is about 37.

On Fig 7, the blue point which is the furthest on the right is from the M0 session of subject 2.

## Analysis of the results and discussion

### Analysis of the results

#### HS/MS and EDSS experiments

The features from the HS/MS experiments (Table 2) quantify what could be observed on Fig 7: the HS and MS groups form clusters with high silhouette scores (over 0.4), but there is some overlap. The HS group is denser that the MS group, which can be explained by the fact that the disease is more severe for some patients than others.

In Fig 8, a global continuity of the color of points can be observed from left (dark blue, low EDSS) to right (dark red, high EDSS). The goal of studying all partitions ({*EDSS* ≤ *i*}, {*EDSS* > *i*}) is to quantify this left/right continuity. The silhouette scores on Table 3 show that {*EDSS* ≤ *i*} and {*EDSS* > *i*} almost always form satisfactory clusters, except for {*EDSS* ≤ *i*} when *i* is above 5 (in that case, the group is sparse and patients with EDSS above 4 are far from the HS). This shows that our method reflects the global progression of the disease by placing MS patients with a low EDSS closer to HS than to patients with a high EDSS.

#### Longitudinal experiment

The objective of this experiment is to study each subject independently from the others to compare their M0 and M6 sessions. The idea behind our approach is that gait signals cannot be compared to an absolute reference but an evolution can be detected by comparing a subject at M6 to himself at M0, thus taking M0 as the reference. Analyzing the signals from subjects with significant values in Table 4 allowed us to highlight three different phenomena:

- A significant change in subject 12’s gait between M0 and M6, which we deduce from the fact that the groups *M*0_12_ and *M*6_12_ are almost completely separable (see Fig 10). This evolution of gait can be linked to the significant evolution of the patient’s disease, as their EDSS goes from 4 to 5 (and is the only one that has a variation of more than 0.5).
- An asymmetrical gait at M6 for subject 15. Usually, the M0 and M6 silhouette scores of a given patient are close because if M0 is separable from M6 then M6 should be easily separable from M0. For subject 15, those scores are *Sil*(*M*0_12_, *M*6_12_) = 0.49 and *Sil*(*M*6_12_, *M*0_12_) = −0.21. This can be explained by the fact that the M6 group is sparser (its MSD and SD are among the highest of all sessions). The M6 group is split in two parts of four points each (see Fig 10). One part (the one closer to the M0 group) is made of the four RF signals of the M6 session, and the other one is made of the LF signals. The significant values for subject 15 on Table 4 can thus be explained by the apparition of an asymmetry at M6. This explanation is supported by clinical evidence.
- An outlier and a technical issue in the signal’s acquisition. Subject 2’s M0 group has a MSD and a SD significantly higher than every other group. It is due to the outlier of the HS group (the blue point on the right of Fig 7), which belongs to *M*0_2_. Visualizing this outlier allowed us, by going back to the associated gait trial, to highlight a segmentation problem during the construction of the database.
  Note that the MSD detects the outlier because the remaining points of the *M*0_2_ group have a low density. If it had been denser the MSD would have been lower but the SD would be similar. This justifies using the squared diameter as a complementary density measure.
  Asymmetry can be detected this way in other patients such as for subject 21, and even, at a lower scale, in the HS group such as for subject 6.

Fig 10 illustrates the above discussion. A similar visual analysis can be performed on all the other subjects using the interactive plots provided with this paper and Table 4.

### Comparison to state of the art

The method presented in this article has the advantage of being non-parametric, except for the two UMAP parameters, for which the default values suggested by the authors of [48] seem to be appropriate. Moreover, it only relies on raw gait signals and does not need additional information such as step annotations or step detection (although, as mentioned before, knowing the number of step for each signal can be useful). Thus, it can be applied to any type of gait-affecting pathology without having to choose new parameters or previously perform step detection or manual annotations.

Our study of multiple sclerosis shows that the method can identify a global correlation between the severity of the disease represented by the EDSS and distance to HS points, and also detects changes in the patients’ gait. Fig 9 shows a point cloud made of dense groups of points that are completely separable from each other. Several groups are made of points with different EDSS scores, and there does not appear to be any way to correlate the distribution of points with their EDSS. This can be explained by the fact that velocity is significantly impacted by other factors than the disease and thus two subjects with different clinical conditions can have the same velocity. In particular, this means that, compared to our approach, the difference of velocity could not be used to detect clinical evolution. Using other standard gait features such as step length, step time (or its variation coefficient) or double stance time (or its variation coefficient) gives similar results to those obtained with velocity.

The use of TDA with sublevel sets to create persistence barcodes and compute distances between them naturally allows to compare signals from the left foot to signals from the right foot, as those barcodes are invariant by translation along the time axis. Allowing comparison between different feet doubles the number of points for each session and thus makes the following analysis more relevant. Moreover, it provides a way to detect asymmetry in a subject’s gait which can further assist clinical gait evaluation. For subjects who have a strong asymmetry, it may be necessary to separate RF and LF signals to study their evolution as the effect of an asymmetry on the distance between points may dominate the effect of any other phenomenon.

### Limitations

This work was limited by the size of the database, that only contains signals from 22 MS patients, and some EDSS values are not represented (4.5 or values under 2). Because of those missing values, we could not study the impact of MS on gait at its earliest stages. Indeed, our study of the ({*EDSS* ≤ *i*} and {*EDSS* > *i*}) groups would allow us to quantify how close patients with low EDSS are to healthy subjects and refine our analysis of the progression of the disease. Having more than two sessions per subject would also be beneficial for the longitudinal study.

The second limitation is the access to a ground truth. We used the EDSS as a measure of the severity of the patients’ disease, but it is limited by its lack of objectivity and of sensitivity to change (and so are other clinical scores for MS) [7–12]. Indeed, in the studied cohort, EDSS scores do not vary by more than 0.5 between M0 and M6 in all cases except one, and often stays the same between two sessions whereas for several patients our method clearly separates the M0 and M6 points. A different ground truth thus seems necessary to compare the results of our method to the conclusions obtained with clinical scores.

### Perspectives

More work may be done to test our method on more patients to study MS or other pathologies including some that involve a left/right asymmetry.

The method can be generalized to analyze different physiological signals or any type of time series, as the only step that is specific to the study of locomotion is the one when bars are removed from persistence barcodes according to the number of steps. Future work may also include using different TDA techniques to improve our method for gait signals or to apply it to other types of signals. An example of such a technique, that is widely used in the literature on TDA for time series (including [28, 29, 35, 36, 38, 39]), is the *delay embedding*, which is a way of transforming a time series into a multi-dimensional point cloud. Using a delay embedding to represent a time series as a *d*-dimensional point cloud allows to study its persistent homology in dimension 0 to *d* − 1 (one persistence barcode can be computed for each dimension), and different dimensions may contain complementary information. A similar approach could also be used to deal with multivariate data. One of the challenges of using a delay embedding is that it makes the method more parametric (it introduces at least two parameters: the dimension of the embedding and the delay) and more difficult to interpret than when using sublevel sets (in which case barcodes can be interpreted in terms of oscillations, as explained above).

## Conclusion

This article has two main contributions: a non-parametric method to study gait signals and visualize the results, and an application to study multiple sclerosis both globally and in a longitudinal way. Our method is based on techniques from topological data analysis, which relies on algebraic topology. Our goal was to present the method in a way that requires no background in topological data analysis to insist on the ideas behind it and make it more easily usable by clinicians.

## Supporting information

S1 File Interactive plot colored by group. Interactive version of the UMAP plot from Fig 7.
(HTML)

S2 File Interactive plot colored by EDSS. Interactive version of the UMAP plot from Fig 8.
(HTML)

S3 File Interactive plot colored by subject ID. Interactive plot where points are colored according to the subjects’ IDs.
(HTML)

S4 File Interactive plot colored by session. Interactive plot where points are colored according to the subjects’ IDs and sessions.
(HTML)

S1 Data (ZIP)
